# Gut-derived genistein from *Parabacteroides distasonis* alleviates psoriatic inflammation via CD200-mediated NF-κB inhibition in mice

**DOI:** 10.1080/19490976.2026.2701386

**Published:** 2026-07-16

**Authors:** Demengjie Le, Mohammad Hassan Heidari, Shunmin Zhu, Yingying Luo, Changzhou Li, Yue Li, Bin Yang, Xuan Wang

**Affiliations:** a Dermatology Hospital, Southern Medical University, Guangzhou, Guangdong, People's Republic of China; b School of Public Health, Southern Medical University, Guangzhou, Guangdong, People's Republic of China; c Department of Plastic and Aesthetic Surgery, Nanfang Hospital of Southern Medical University, Guangzhou, Guangdong, People's Republic of China

**Keywords:** Gut–skin axis, parabacteroides distasonis, genistein, CD200, psoriasis

## Abstract

The gut-skin axis plays a pivotal role in psoriasis pathogenesis, yet the precise metabolic crosstalk by which intestinal commensals regulate cutaneous immunity remains elusive. Here, we identify a functional “microbe-enzyme-metabolite-immune” axis that orchestrates skin homeostasis. Through multi-omics analyses of psoriatic patients and imiquimod-induced murine models, we reveal a significant depletion of the gut commensal *Parabacteroides distasonis* (*P. distasonis*) and its metabolite, genistein. We demonstrate that *P. distasonis* utilizes its inherent β-glucosidase activity to convert dietary genistin into bioactive genistein, a process we validated using an engineered β-glucosidase-expressing *E. coli* strain. Therapeutically, supplementation with *P. distasonis* or genistein significantly ameliorates psoriatic phenotypes, restores skin barrier integrity (filaggrin/loricrin), and suppresses IL-23/IL-17-mediated inflammation. Mechanistically, we uncovered that genistein enhanced CD200-CD200R signaling and suppressed macrophage activation. It effectively reactivates CD200 expression, thereby inhibiting the canonical NF-κB signaling pathway and blunting macrophage-driven inflammation. Notably, the therapeutic efficacy of this axis was abrogated by CD200 blockade, confirming its indispensability. Collectively, our findings elucidate a causal mechanism linking gut microbial enzyme activity to host skin immunity, highlighting *P. distasonis*-derived genistein as a promising precision intervention for psoriasis management.

## Highlights

- *Parabacteroides distasonis* is significantly depleted in psoriatic patients and mice.
- *P. distasonis*-derived β-glucosidase converts genistin into bioactive genistein.
- Activation of the CD200-CD200R axis inhibits NF-κB signaling and restores skin barrier integrity.
- The study establishes a novel “microbe-enzyme-metabolite-immune” therapeutic axis.

## Graphical abstract

**Figure:**
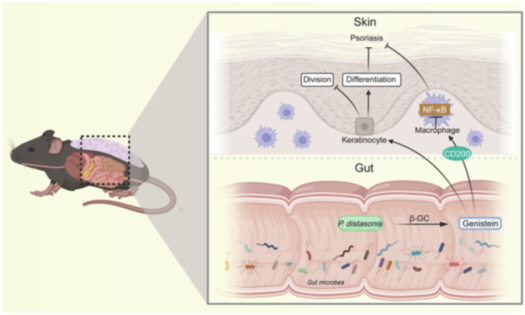

Psoriasis disrupts the balance of gut microbes, characterized by a significant decrease in the abundance of *P. distasonis,* accompanied by a reduction in genistein levels. Genistein is generated via the hydrolysis of genistin by β-glucosidase (β-GC) in *P. distasonis*. Genistein suppresses inflammatory cytokine release from macrophages by upregulating *Cd200* expression. Additionally, genistein promotes keratinocyte differentiation while inhibiting their proliferation.

## Introduction

Psoriasis is a chronic immune-mediated disorder that primarily affects the skin and joints, with interleukin (IL) -17 A and IL-23 recognized as pivotal drivers of its pathogenesis.^1^ ^,^ ^2^ It can manifest at any age and imposes a considerable burden on patients‘ quality of life and health-care systems.^3^ Due to its relapsing nature, effective management of psoriasis requires a multifaceted approach, combining pharmacological therapies with lifestyle modifications and personalized interventions.^1^ Emerging evidence suggests that gut microbiota plays a critical role in modulating systemic and cutaneous immune responses. In particular, dietary interventions or probiotic supplementation have demonstrated promising benefits in immune-mediated skin diseases, including psoriasis.^4‐6^ The so-called *gut-skin* axis, which describes the bidirectional communication between intestinal microbes and skin physiology, has become a focal point of dermatological research. For example, microbial -derived short-chain fatty acids(SCFAs) enhance skin barrier integrity by modulating mitochondrial metabolism in keratinocytes.^7^ Similarly, *Bifidobacterium longum* alleviates atopic dermatitis by metabolizing tryptophan into aryl hydrocarbon receptor (AHR) ligands, thereby regulating cutaneous inflammation.^8^ In psoriasis, parallel studies have revealed microbial dysbiosis and correlations between specific gut taxa and disease severity.^4,9‐11^ However, the precise microbial metabolites and downstream immune pathways linking gut microbiota to psoriatic inflammation remain poorly defined.

Soy isoflavones are a class of naturally occurring polyphenolic compounds abundantly present in legumes such as soybeans. These phytochemicals exhibit a wide range of pharmacological activities, including antioxidant, anti-inflammatory, and immunomodulatory effects.^12‐14^ In their dietary form, isoflavones predominantly exist as glycoside conjugates, which are biologically inactive until hydrolyzed into their aglycone forms-namely genistein (GEN), daidzein (DA), and glycitein (GLY).^15^ This hydrolysis is primarily catalyzed by microbial enzymes such as β-glucosidase (β-GC) or β-galactosidase (β-GAL), highlighting the essential role of gut microbiota in regulation isoflavone bioavailability and activity.^16‐18^ Notably, recent studies have demonstrated that formononetin, a representative isoflavone aglycone, can alleviate psoriatic skin inflammation and keratinocyte hyperproliferation by suppressing IL-17 and IL-23 production and inhibiting the NF-κB signaling pathway.^19^ While current evidence underscores the therapeutic potential of isoflavones in managing inflammatory skin diseases, the mechanistic interplay between gut microbiota-dependent metabolic pathways and their downstream immune effects in psoriasis remain largely unexplored.

While the IL-23/IL-17 axis is a primary therapeutic target, the psoriatic lesion microenvironment is characterized by a complex and dense infiltrate of diverse immune cell populations. Macrophages are predominantly aggregated along the dermal-epidermal junction, where they contribute significantly to local inflammation.^20^ Macrophages secrete pro-inflammatory cytokines such as tumor necrosis factor-α (TNF-α) and IL-6, which play critical roles in regulating keratinocyte proliferation and differentiation.^21^ As such, targeting macrophage-driven inflammation represents a promising strategy to ameliorate cutaneous abnormalities associated with psoriasis.^22^ ^,^ ^23^ Despite increasing interest in microbiota–immune interactions, the specific crosstalk between intestinal microbes, microbial metabolites, and macrophage-driven inflammation in psoriasis remains poorly defined.

To address this gap, the present study investigates the regulatory role of the gut–skin axis in a murine model of imiquimod-induced psoriasiform dermatitis. We identify a functional microbiota–metabolite–immune signaling axis through which the gut commensal *Parabacteroides distasonis* ameliorates skin inflammation. Mechanistically, *P. distasonis* utilizes its β-glucosidase activity to convert dietary genistin into bioactive genistein. These findings offer new mechanistic insight into how gut commensals regulate distant skin immune responses and highlight the therapeutic potential of microbiota-guided natural product interventions for immune-mediated skin disorders.

## Results

### Gut dysbiosis exacerbates psoriatic skin lesions by impairing epidermal barrier and promoting IL-23/Th17 inflammation

**Figure 1.**
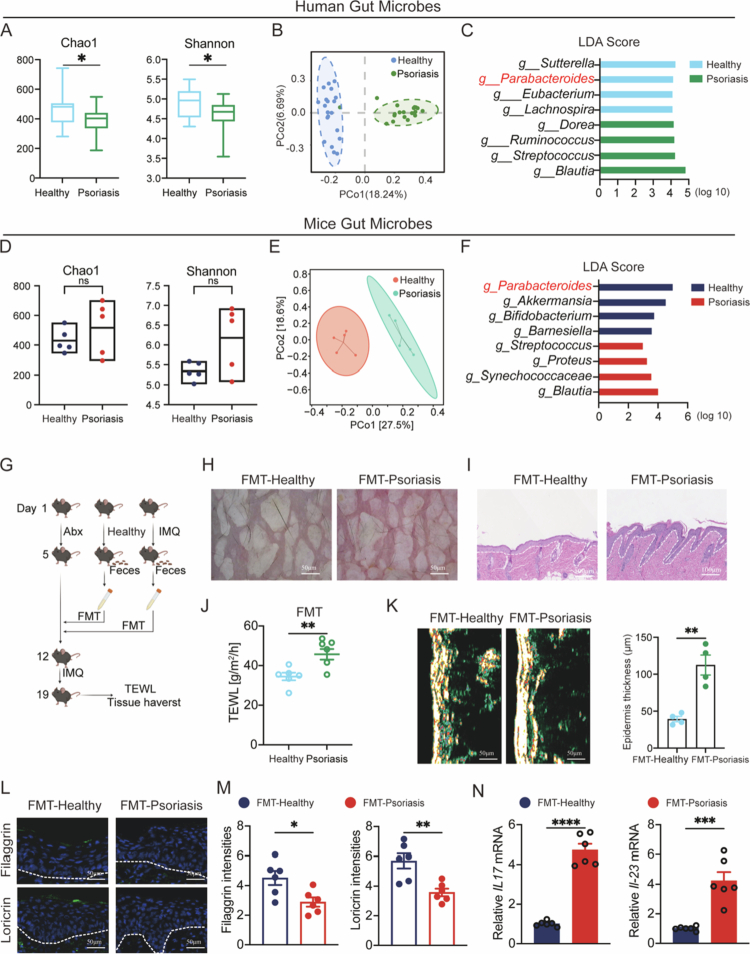
Gut dysbiosis exacerbates psoriatic skin lesions by impairing epidermal barrier and promoting IL-23/Th17 inflammation. (A) Alpha diversity analysis of gut microbiota in psoriasis patients, assessed by Chao1 and Shannon indices. (B) Beta diversity visualized by principal coordinate analysis (PCoA) based on Bray-Curtis dissimilarity. (C) LEfSe analysis showed differences in bacterial profiles among healthy individuals and psoriasis patients. (D) Alpha diversity assessment in healthy and IMQ-treated mice (*n* = 5 per group). (E) PCoA plot of gut microbial communities in IMQ-treated and healthy mice based on Bray-Curtis dissimilarity. (F) LEfSe analysis showed differences in bacterial profiles in IMQ-treated mice. (G) Schematic diagram illustrates the fecal microbiota transplantation (FMT) procedure following antibiotic (Abx) pretreatment (*n* = 6 per group). (H) Dermoscopic evaluation of dorsal skin post-FMT. (I) Representative hematoxylin and eosin (H&E) staining of skin sections. (J) Measurement. of transepidermal water loss (TEWL). (K) High-frequency ultrasound imaging of epidermal thickness. (L, M) Immunofluorescence staining and quantification of skin filaggrin and loricrin expression in skin tissue. (N) qRT-PCR quantification of inflammatory cytokines (*Il-17, Il-23*). ns, *P* > 0.05; *P < 0.05; **P < 0.01; ***P < 0.001; ****P < 0.0001.

To investigate the influence of gut microbiota on psoriatic skin lesions, we first analyzed microbiome alterations during psoriasis progression. Reanalysis of publicly available 16S rRNA datasets from psoriatic patients^4^ revealed significantly lower alpha diversity in psoriatic patients compared to healthy group (Figure 1A). Principal coordinate analysis (PCoA) based on Bray-Curtis dissimilarity showed significant separation between groups (Figure 1B), with concomitant changes in microbial composition at the genus level (Figure 1C).

We then established an imiquimod (IMQ)-induced psoriatic mouse model (Figure S1A). Following 7-day IMQ exposure, mice developed characteristic psoriatic phenotypes including dorsal scaling, pronounced epidermal hyperplasia (Figures S1B, S1C and S1E), and elevated transepidermal water loss (TEWL; Figure S1D). These pathological changes correlated with reduced expression of epidermal barrier proteins (filaggrin and loricrin; Figures S1F, S1G, and S1I), and significantly elevated levels of proinflammatory cytokines such as (IL-17A, IL-23, TNF-α, and IL-1β) in skin tissues (Figure S1H). 16S rRNA sequencing of fecal microbiota revealed no significant difference in alpha diversity between IMQ-treated and healthy groups (Figure 1D). In contrast, beta diversity demonstrated significant dissimilarity in community structures (Figure 1E), along with corresponding alterations in taxonomic composition (Figure 1F).

To delineate the functional impact of gut microbiota dysbiosis on psoriasis pathogenesis, antibiotic-pretreated mice received fecal microbiota transplantation (FMT) from IMQ-treated or healthy donors (Figure 1G). Mice receiving gut microbiota from IMQ-treated donors developed exacerbated psoriatic skin lesions (Figure 1H), with marked epidermal hyperplasia (Figure 1I and 1K) and elevated TEWL (Figure 1J). These changes were accompanied by downregulation of epidermal barrier proteins (Figure 1L and 1M), as well as increased expression of IL-17A and IL-23 mRNA levels in skin tissue (Figure 1N).

Collectively, these findings indicate that gut dysbiosis directly aggravates psoriatic pathology by impairing cutaneous barrier integrity and amplifying IL-23/Th17-mediated inflammation.

### Exogenous administration of *P. distasonis* improves epidermal permeability barrier function

**Figure 2.**
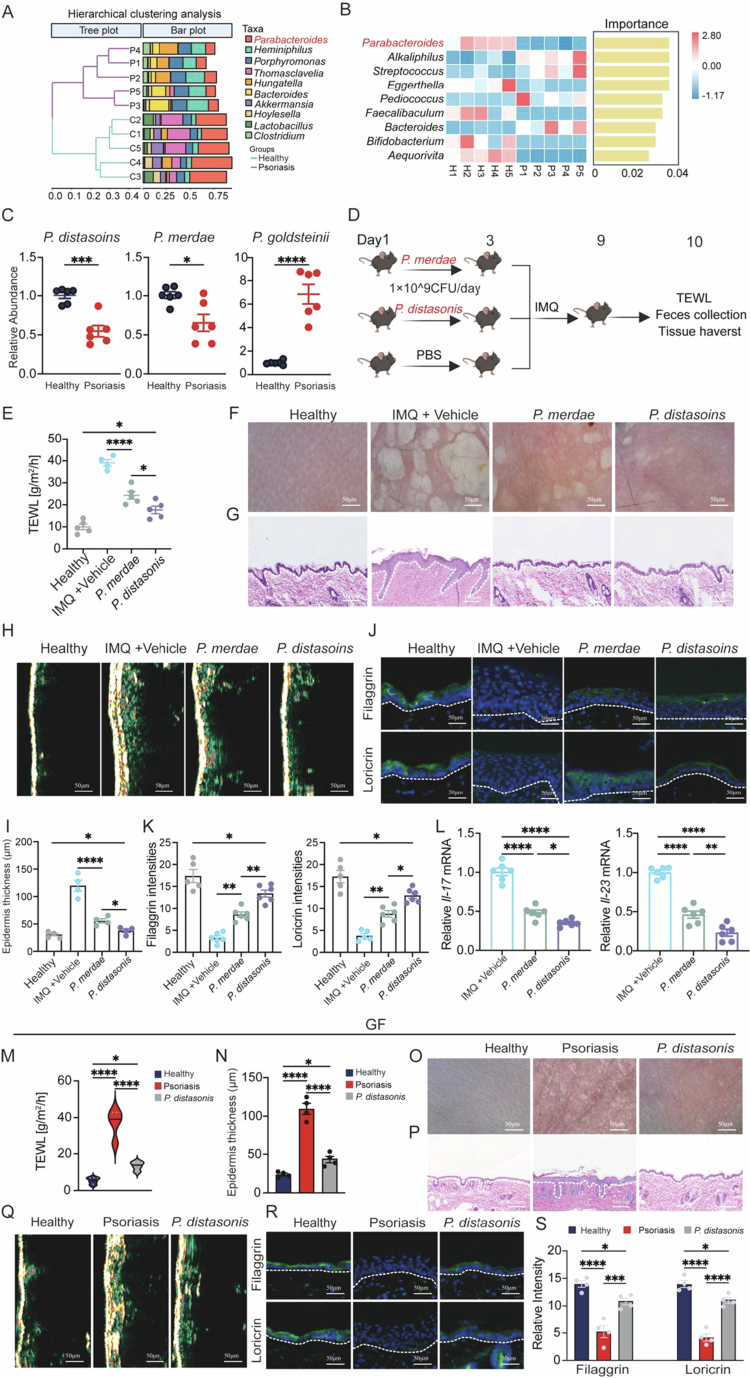
Exogenous administration of *P. distasonis* improves epidermal permeability barrier function. (A) Hierarchical clustering of gut microbiota in IMQ-treated and healthy mice (*n* = 5 per group). (B) Machine learning-based identification of gut microbes’ compositional variations in psoriatic mice (*n* = 5 per group). (C) Relative abundance of *Parabacteroides* species (*P. distasonis*, *P. merdae*, *P. goldsteinii*) in IMQ-treated and healthy mice (*n* = 6 per group). (D) Experimental design showing pretreatment with *P. distasonis* and *P. merdae* before IMQ application (*n* = 6 per group). (E) Quantification of transepidermal water loss (TEWL) following bacterial administration. (F) Representative dermoscopic images of dorsal skin lesions after pretreatment. (G) Representative H&E staining of skin sections. (H) High-frequency ultrasound imaging of dorsal skin lesions. (I) Quantification of epidermal thickness based on ultrasound analysis. (J) Representative immunofluorescence staining of filaggrin and loricrin in lesional skin sections. (K) Quantitative analysis of filaggrin and loricrin fluorescence intensity. (L) Relative mRNA expression levels of *Il17a* and *Il23* in skin tissues after bacterial intervention. (M) TEWL measurements in germ-free (GF) mice subjected to mono-colonization with *P. distasonis*. (N) Quantification of epidermal thickness in GF mice. (O and P) Representative dermoscopic images (O) and H&E staining (P) of dorsal skin lesions in GF mice. (Q) High-frequency ultrasound imaging of dorsal skin lesions in GF mice. (R) Representative immunofluorescence staining of filaggrin and loricrin in skin sections from GF mice. (S) Quantitative analysis of filaggrin and loricrin fluorescence intensity in GF mice. *P < 0.05; **P < 0.01; ***P < 0.001; ****P < 0.0001.

Initial linear discriminant analysis effect size (LEfSe) of the gut microbiome identified a concurrent depletion of the genus *Parabacteroides* in both psoriasis patients and an IMQ-induced murine model (Figure 1C and 1F). To elucidate the microbial taxa most critical to skin barrier integrity in the IMQ-treated mice, we employed both hierarchical clustering and machine learning algorithms. These computational approaches underscored the prominence of *Parabacteroides* in the context of murine psoriatic inflammation (Figure 2A and 2B). This finding was consistent with our human data, where integrated 16S rRNA sequencing corroborated the significant depletion of *Parabacteroides* in the gut of psoriasis patients (**Figures S2B** and **S2C**).

*Parabacteroides* is recognized as a probiotic with potential therapeutic applications in various diseases.^24‐26^ To elucidate the specific functional roles of the altered bacterial composition in the pathogenesis of psoriasis, we focused on *P. distasonis*, *P. merdae*, and *P. goldsteinii*. These three species were selected for *in vivo* functional screening because they constitute the most dominant and abundant members of the *Parabacteroides* genus in both human and murine gut microbiomes. Furthermore, our species-level profiling revealed distinct dynamic shifts among them during IMQ induction: *P. distasonis* and *P. merdae* were significantly depleted, whereas *P. goldsteinii* was concomitantly enriched (**Figures S2A** and **2C**). Therefore, we pretreated mice with these representative strains (without prior antibiotic clearance) two days prior to the treatment with IMQ (Figure 2D). Persistent detection of *P. distasonis* in mouse fecal samples upon experiment termination confirms successful bacterial colonization (**Figure S2F**). The results showed that pretreatment with *P. distasonis* exhibited a more potent effect than *P. merdae* in decreasing TEWL, erythema, and scaling, accompanied by a greater reduction in epidermal thickness (Figures 2E-2I). Furthermore, pretreatment with *P. distasonis* led to a more pronounced upregulation of differentiation marker-related proteins in IMQ-treated mice (Figures 2J, 2K, **S2E,** and **S2G**), while suppressing the inflammatory response (Figure 2L). Accordingly, subsequent experiments mainly explored the function of *P. distasonis* in skin damage of mice with psoriasis.

Meanwhile, we verified the above findings in germ-free (GF) mice. The results showed that *P. distasonis* pretreatment profoundly alleviated IMQ-induced psoriasis-like skin lesions in the absence of commensal microbiota. *P. distasonis* significantly decreased transepidermal water loss (TEWL) to a level comparable to healthy group (Figure 2M). Dermoscopic observations and H&E staining images revealed that *P. distasonis* substantially ameliorated skin lesions (Figures 2O and 2P). Consistently, ultrasonography (Figure 2Q) and histological quantification (Figure 2N) further demonstrated that *P. distasonis* effectively suppressed IMQ-induced epidermal hyperplasia. At the molecular level, immunofluorescence staining indicated that IMQ application drastically reduced the expression of key skin barrier proteins, filaggrin and loricrin. However, pretreatment with *P. distasonis* successfully rescued the expression of both proteins (Figure 2R), and the quantitative relative intensities showed no statistical difference compared to the healthy group (Figure 2S). Collectively, our data reveal a significant reduction of intestinal *P. distasonis* in mice treated with imiquimod, providing a strong rationale for exploring the therapeutic potential of its exogenous administration in mitigating psoriatic inflammation.

### Genistein improves epidermal permeability barrier function in IMQ-treated mice

**Figure 3.**
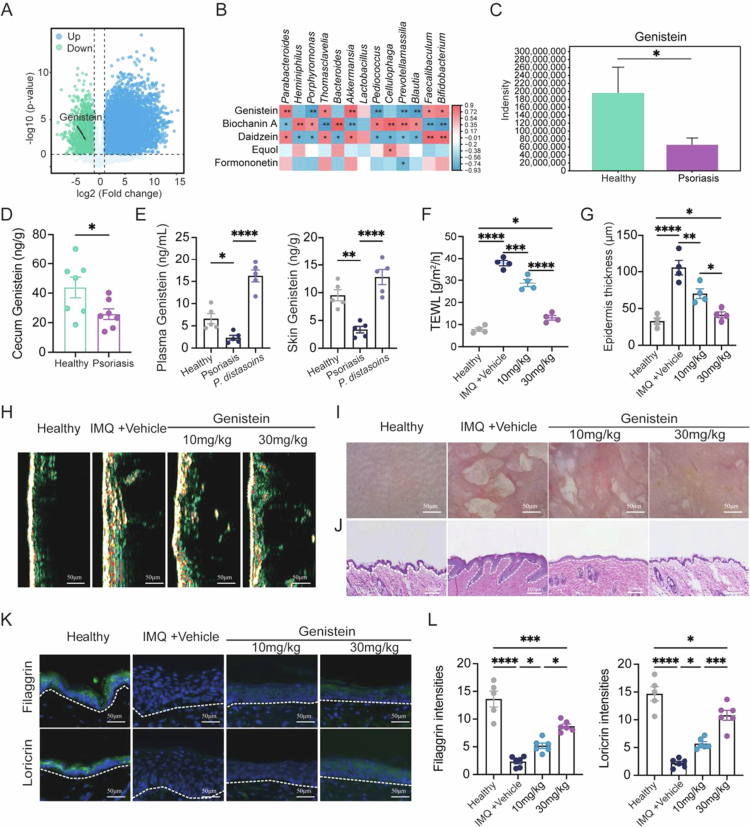
Genistein improves epidermal permeability barrier function in IMQ-treated mice. (A) Volcano plot analysis revealed significant downregulation of genistein in IMQ-induced psoriatic mice compared to healthy group (*n* = 5 per group). (B) Correlation analysis between isoflavonoids and gut microbiota composition. (C, D) Fecal genistein content analysis in psoriatic mice. (E) Plasma and skin genistein levels in *P. distasonis*-pretreated psoriatic mice. (F) Transepidermal water loss (TEWL) measurements of dorsal skin in genistein-pretreated psoriatic mice (*n* = 4 per group). (G) Quantification of epidermis thickness of dorsal skin in the indicated groups. (H) Representative ultrasonography images of dorsal skin in genistein-pretreated psoriatic mice. (I) Dermoscopy examination of dorsal skin lesions. (J) Representative H&E staining images of dorsal skin sections. (K) Immunofluorescence staining of skin barrier proteins (filaggrin and loricrin) in the dorsal skin. (L) Statistical analysis of filaggrin and loricrin fluorescence intensities in the indicated groups (*n* = 6 per group). *P < 0.05; **P < 0.01; ****P < 0.0001.

Given the pronounced ameliorative effects of *P. distasonis* on psoriatic lesions, we hypothesized that its protective actions are mediated through specific metabolic alterations. This led us to broaden our inquiry from a single organism to the wider metabolic consequences of gut microbial dysbiosis. We therefore proceeded to interrogate how aberrant gut microbial metabolism contributes to the heightened inflammatory response in the skin of IMQ-treated mice. Untargeted metabolomics of fecal samples revealed significant compositional differences in metabolites between IMQ-treated and healthy mice (**Figure S3A**). Machine learning analysis further pointed to the loss of genistein as a key driver in the progression of psoriasis (**Figure S3B**). Volcano plot and heatmap analyses consistently confirmed the reduced levels of genistein in IMQ-treated mice (Figure 3A and **S3C**). Subsequent correlation studies with various isoflavones revealed that genistein exhibited the strongest association with *Parabacteroides* abundance among all tested compounds (Figure 3B). LC-MS quantification validated the specific reduction of fecal genistein in IMQ-treated mice (Figure 3C and 3D). *P. distasonis* pretreatment obviously raised genistein concentrations in plasma and skin tissues (Figure 3E).

To assess the therapeutic potential of genistein, mice were pretreated with genistein via intraperitoneal injection prior to IMQ induction. TEWL measurements demonstrated that genistein significantly attenuated IMQ-treated TEWL, with higher concentrations yielding more pronounced protective effects (Figure 3F). Dermoscopic analysis revealed reduced scaling on the dorsal skin of genistein-pretreated IMQ-treated mice (Figure 3G). Furthermore, genistein pretreatment markedly ameliorated epidermal thickening and enhanced the expression of skin barrier proteins (Figure 3H–3L and **S3D**). Concurrently, inflammatory cytokine levels in skin tissues were significantly reduced following pretreatment with higher genistein concentrations (**Figure S3E**). Collectively, these findings provide compelling evidence that psoriatic progression correlates with decreased genistein levels, while exogenous genistein supplementation effectively mitigates IMQ-treated cutaneous damage.

### *P. distasonis* facilitates genistein release via β-glucosidase activity

**Figure 4.**
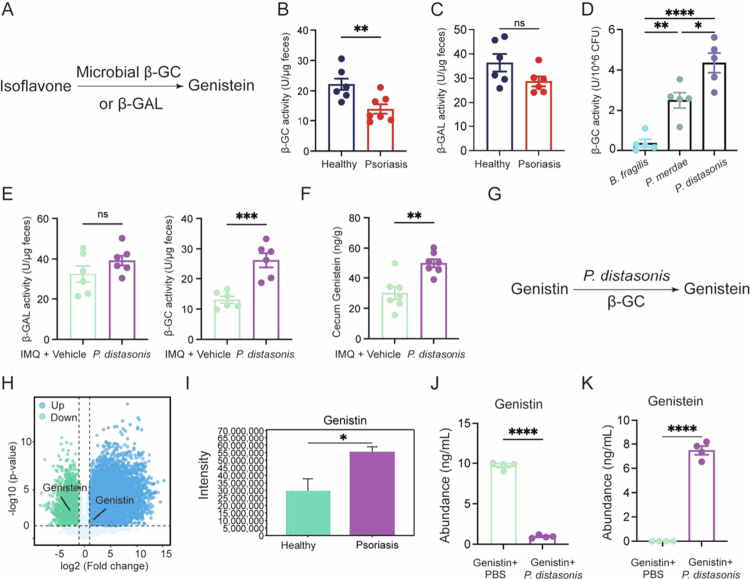
*P. distasonis* facilitates genistein release via β-glucosidase activity. (A) Microbial β-glucosidase or β-galactosidase is required to convert isoflavones into bioactive free forms (genistein). (B, C) Fecal enzymatic activities of β-glucosidase and β-galactosidase in IMQ-treated mice (*n* = 6 per group). (D) β-glucosidase activity across distinct bacterial strains (*n* = 5 replicates). (E) Fecal β-glucosidase and β-galactosidase activities in IMQ-treated mice following *P. distasonis* gavage administration (*n* = 6 mice per group). (F) Quantification of fecal genistein levels in *P. distasonis-*treated psoriatic mice (*n* = 7 per group). (G) Proposed metabolic pathway: β-glucosidase catalyzes the conversion of conjugated genistin to free genistein. (H) Volcano plot analysis of fecal metabolome reveals differential abundance of genistin and genistein in IMQ-treated mice. (I) Quantitative analysis of fecal genistin levels in IMQ-treated mice. (J, K) Genistin consumption and genistein production in *P. distasonis* co-culture systems with genistin substrate (*n* = 4 per group). ns, *P* > 0.05; *P < 0.05; **P < 0.01; ***P < 0.001; ****P < 0.0001.

The above results demonstrate that genistein exhibits the strongest positive correlation with *Parabacteroides* abundance in IMQ-treated mice (Figure 3B). To investigate whether *P. distasonis* mediates genistein release, we focused on microbial β-glucosidase (β-GC) and β-galactosidase (β-GAL), which are key enzymes responsible for the hydrolysis of isoflavone^27^ ^,^ ^28^ (Figure 4A). Fecal enzyme analysis revealed a significan reduction in β-GC activity, but not in β-GAL activity, in IMQ-treated mice (Figure 4B and 4C). Notably, *P. distasonis* exhibited the highest β-GC activity among tested microbes (Figure 4D). Restoration of β-GC activity and concomitant elevation of fecal genistein levels were observed in *P. distasonis*-pretreated IMQ mice (Figure 4E and 4F). These results suggest that *P. distasonis* mediated the release of genistein.

Considering genistein's origin from genistin conversion,^29^ we hypothesized that *P. distasonis* facilitates β-GC-mediated genistein liberation from its precursor (Figure 4G). Metabolomic analysis revealed reciprocal changes in the levels of genistin and genistein in fecal samples from IMQ-treated mice (Figure 4H and 4I), supporting this hypothesis. To confirm these findings, an *in vitro* experiment was conducted to evaluate the release profile of genistin. Co-incubation of *P. distasonis* with genistin for 24 hours resulted in a marked increase in genistein levels in the culture supernatant, indicating that *P. distasonis* can efficiently convert genistin to genistein (Figure 4J and 4K). Taken together, these results suggest that *P. distasonis* exerts cutaneous protective effects in IMQ-treated psoriatic mice via the release of genistein.

### β-glucosidase mediates the genistein-dependent protective effects of *P. distasonis*

**Figure 5.**
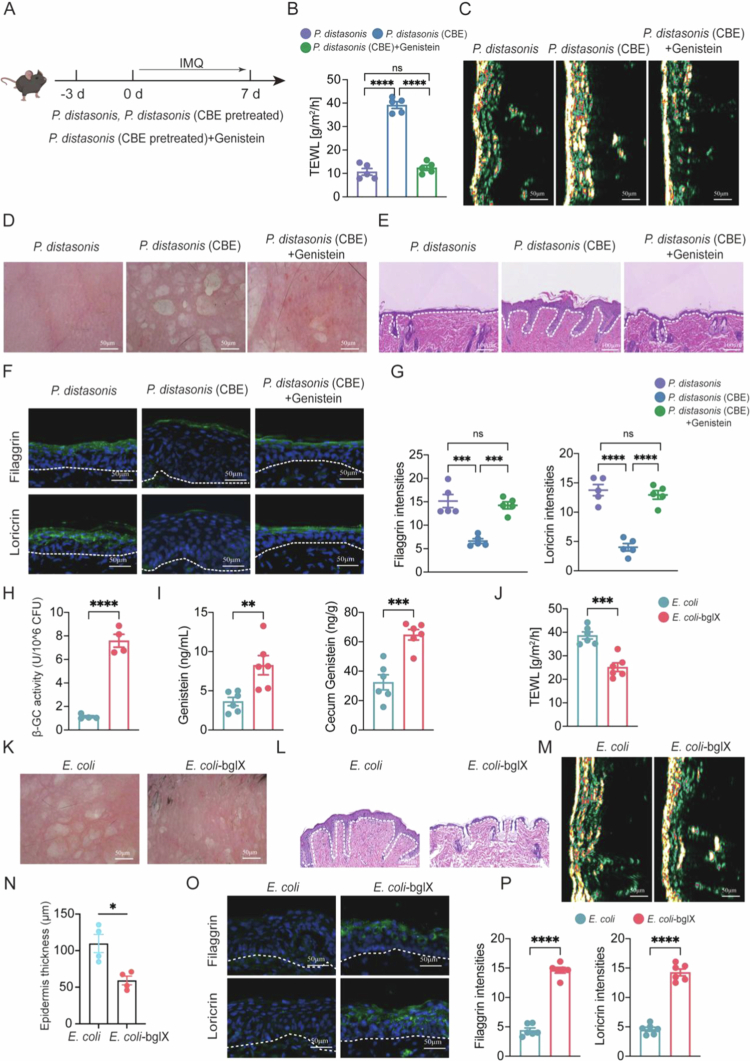
β-glucosidase mediates the genistein-dependent protective effects of *P*. distasonis. (A) Experimental design of IMQ-induced psoriatic mice following β-glucosidase inhibition in *P. distasonis* strain using CBE (*n* = 5 per group). (B) TEWL measurements in mice receiving *P. distasonis* with or without CBE pretreatment. (C) Epidermal ultrasonography analysis. (D) Dermatoscopic evaluation of skin lesions. (E) Histopathological assessment by H&E staining. (F-G) Skin tissue immunofluorescence staining and quantitative analysis. (H) Comparative β-glucosidase activity between wild-type *E. coli* and bglX-deficient strains (*n* = 4 replicates). (I) Genistein production capacity of *E. coli-*bglX and fecal genistein levels in gavaged psoriatic mice (*n* = 6 per group). (J) TEWL measurements in *E. coli-*bglX-treated psoriatic mice. (K) Dermoscopy examination of dorsal skin in *E. coli-*bglX-treated psoriatic mice. (L) H&E staining in *E. coli-*bglX-treated psoriatic mice. (M-N) Ultrasonographic evaluation of epidermal structure and quantification of epidermis thickness in *E. coli-*bglX-treated psoriatic mice. (O-P) Immunofluorescence visualization and quantification in *E. coli-*bglX-treated psoriatic mice. (*n* = 6 per group). ns, *P* > 0.05; **P < 0.01; ***P < 0.001; ****P < 0.0001.

Building upon our previous findings regarding the alterations of β-glucosidase activity and genistein levels upon *P. distasonis* pretreatment, we further validated the protective role of the *P. distasonis*-β-glucosidase-genistein axis in psoriatic skin lesions through *in vivo* experiments. Mice were pretreated with either *P. distasonis*, β-galactosidase inhibitor Conduritol B Epoxide (CBE)-pretreated *P. distasonis*, or genistein-supplemented regimens prior to IMQ treatment (Figure 5A). Notably, CBE-pretreated *P. distasonis* lost its protective effects on the skin barrier, as evidenced by elevated TEWL, increased epidermal thickness, enhanced scaling severity (Figure 5B–5E), and reduced expression of skin barrier-related proteins (Figure 5F and 5G). Furthermore, CBE-pretreated *P. distasonis* failed to suppress the IMQ-induced expression of proinflammatory cytokines (**Figure S4A**).

To further validate the critical role of β-glucosidase in mediating genistein release from *P. distasonis*, we engineered a β-glucosidase-expressing strain. Initially, we identified the β-glucosidase-encoding gene (*bgl-X*) in the *P. distasonis* genome (Figure S4B) and integrated it into *E. coli* Nissle 1917 via homologous recombination, generating the *E. coli-bgl-X* strain (Figure S4C). The growth rate of *E. coli* remained unchanged after this genetic modification (Figure S4D). Enzymatic assays confirmed significantly elevated β-glucosidase activity in *E. coli-bgl-X* compared to the native *E. coli* strain (Figure 5H), verifying successful engineering. Both *in vitro* and *in vivo* experiments demonstrated that *E. coli-bgl-X* markedly increased genistein levels (Figure 5I), confirming the pivotal role of β-glucosidase in genistein liberation. Notably, administration of *E. coli-bgl-X* in IMQ-treated mice significantly attenuated TEWL (Figure 5J), reduced epidermal scaling and thickness, and enhanced skin barrier protein expression (Figures 5K–5O). Concurrently, it suppressed cutaneous inflammatory cytokine levels (**Figure S4E**). These findings collectively demonstrate that the *P. distasonis*-β-glucosidase-genistein metabolic axis critically preserves skin barrier integrity in IMQ-treated mice.

### Genistein attenuates macrophage-mediated inflammation by upregulating CD200-CD200R axis

**Figure 6.**
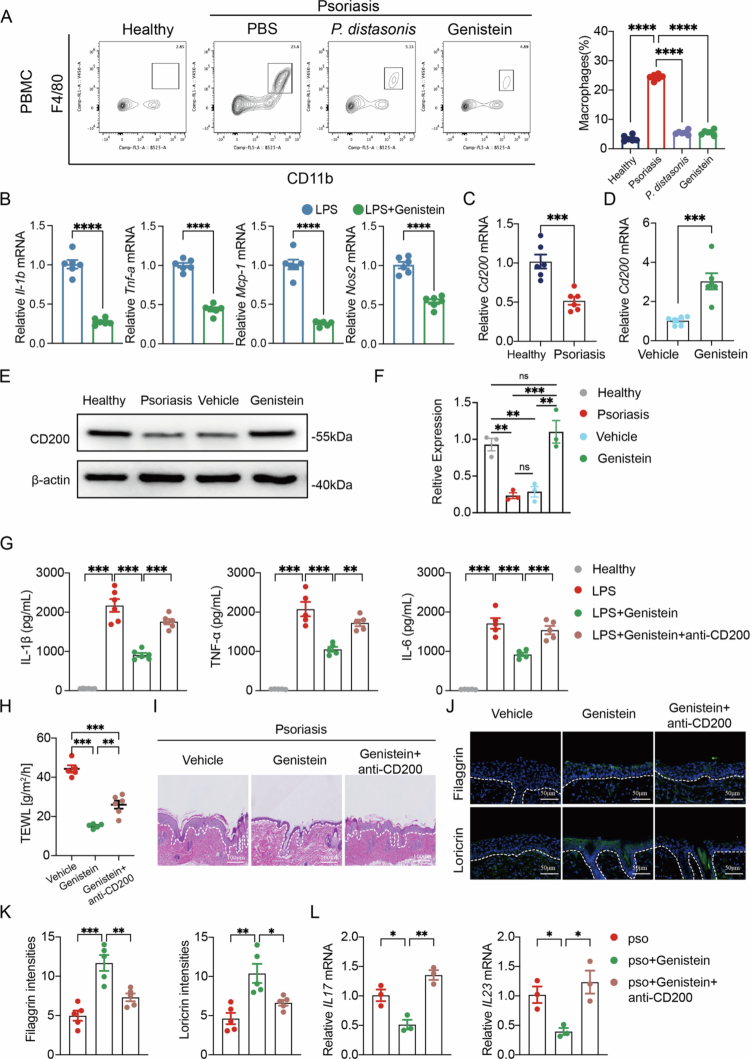
Genistein attenuates macrophage-mediated inflammation by upregulating CD200. (A) Flow cytometric quantification of circulating macrophages in psoriatic mice (*n* = 6 per group). (B) qRT-PCR analysis of inflammatory cytokine mRNA levels in genistein-treated macrophages *in vitro* (*n* = 6 per group). (C–F) qRT-PCR (C and D) and Western blot (E and F) analysis of CD200 levels in the skin of IMQ-treated mice and genistein-treated mice. (G) BMDMs were induced with LPS (1 μg/mL), or treated with LPS (1 μg/mL) + genistein (10 μM), or preincubated with 10 μg/mL anti-CD200 antibody for 30 min followed by LPS (1 μg/mL) + genistein (10 μM) treatment. IlL-1β, TNF-α, and IL-6 levels in the cell supernatants were then measured. (H–L) During the IMQ-treated period, mice were daily intraperitoneally injected with genistein (10 mg/kg) or intraperitoneally injected with genistein (10 mg/kg) + anti-CD200 antibody (200 μg/mouse). TEWL (H), epidermal thickness of the dorsal skin (I), tissue immunofluorescence staining of skin barrier proteins (J and K), and expression of skin tissue inflammatory factors (L) after neutralization of CD200 were shown. (*n* = 3–6 per group). **P < 0.01, ***P < 0.001, ****P < 0.0001.

To explore the mechanisms underlying genistein-mediated skin barrier protection in IMQ-induced psoriatic mice, we focused on its anti-inflammatory effects, particularly on macrophage activation. Flow cytometry and histological analysis revealed that both *P. distasonis* and genistein significantly reduced macrophage infiltration in the blood and lesional skin (Figures 6A, S5A, and S5B). *In vitro*, co-culture of genistein with macrophages markedly suppressed the expression of pro-inflammatory cytokines, confirming direct anti-inflammatory effects (Figure 6B).

Considering that the CD200-CD200R axis plays a pivotal role in negatively regulating macrophage activity upon microbial or viral stimulation, we next assessed CD200 expression. IMQ-treated mice exhibited significantly reduced *Cd200* mRNA (Figure 6C and 6D) and protein expression in skin tissues, whereas genistein administration restored CD200 levels (Figure 6E and 6F). To further confirm the involvement of CD200, a blocking anti-CD200 antibody was introduced in the genistein-macrophage co-culture system, which reversed the inhibitory effects of genistein on inflammatory cytokine secretion (Figure 6G) Similarly, antibody blockade *in vivo* abolished the genistein-induced reduction in TEWL (Figure 6H), and failed to improve epidermal hyperplasia or upregulate barrier-associated proteins (Figures 6I, 6J and 6K).

Moreover, CD200 blockade abrogated the anti-inflammatory effects of genistein in vivo, resulting in elevated cutaneous levels of IL-1β, TNF-α, and IL-6 (Figure 6L). These findings collectively demonstrate that genistein alleviates psoriasis-associated inflammation by reactivating CD200 expression, thereby suppressing macrophage-mediated inflammatory responses.

### Genistein suppresses macrophage inflammation via the NF-κB signaling pathway and modulates keratinocyte behavior

**Figure 7.**
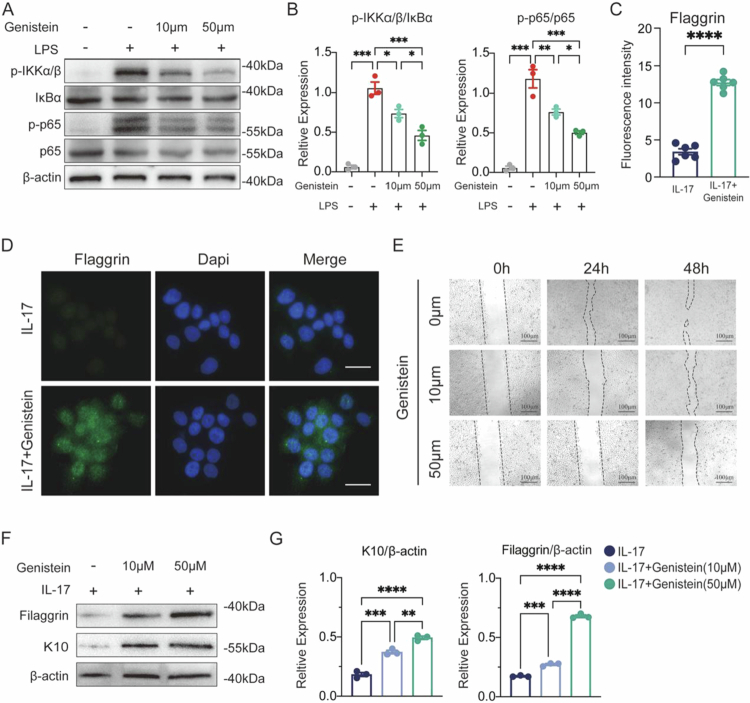
Genistein suppresses macrophage inflammation via the NF-κB signaling pathway and modulates keratinocyte behavior. (A-B) BMDMs were induced with LPS (1 μg/mL) and treated without or with genistein (10 μM or 50 μM), followed by Western blot analysis of the NF-κB signaling pathway. (*n* = 3 per group). (C-D) Immunofluorescence imaging of genistein-treated keratinocytes (*n* = 6 per group). (E) Scratch wound assay in keratinocytes treated with genistein (10 μM or 50 μM) or vehicle. (F, G) Western blot detection of differentiation markers (K10, Filaggrin) in genistein-treated keratinocytes. (*n* = 3 per group). *P < 0.05; **P < 0.01; ***P < 0.001; ****P < 0.0001.

Given that CD200 negatively regulates NF-κB signaling, we further examined whether genistein modulates this pathway to exert its anti-inflammatory effects. Western blot analysis demonstrated that genistein significantly inhibited LPS-induced phosphorylation of IκBα and p65 in macrophages (Figure 7A and 7B), indicating suppression of the canonical NF-κB signaling cascade. In addition to its immunomodulatory effects, genistein also influenced epidermal cell homeostasis. Specifically, genistein enhanced the expression of keratinocyte differentiation markers, including filaggrin and keratin 10 (K10), as shown by immunoblotting and immunofluorescence staining (Figures 7C, 7D, 7F, and 7G) Meanwhile, genistein treatment markedly suppressed keratinocyte proliferation (Figure 7E). Collectively, these results indicate that genistein exerts anti-inflammatory effects through NF-κB inhibition in macrophages and restores epidermal barrier integrity by enhancing keratinocyte differentiation and limiting hyperproliferation.

## Discussion

Alterations in gut microbiota are associated with susceptibility to various human diseases, including psoriasis.^6^ ^,^ ^30^ ^,^ ^31^ Although consistent evidence reveals gut microbial dysbiosis in psoriasis patients,^32^ the causal relationship between these microbial shifts and the disease development is not yet fully elucidated. Cantabrana et al. reported that psoriatic plaques exhibited the highest microbial diversity, with *Firmicutes* predominating, leading to an elevated *Firmicutes*/*Bacteroidetes* ratio.^4^ Similarly, Stehlikova et al. suggested that skin inflammation in psoriasis may be directly mediated by the intestinal microbiome, emphasizing the potential role of gut-skin axis in the pathogenesis of psoriasis.^33^ By integrating microbiome sequencing with metabolomics and functional validation, we identified *Parabacteroides distasonis* as a key commensal whose β-glucosidase–mediated metabolism of dietary isoflavones produces genistein, a metabolite with direct effects on keratinocyte differentiation and macrophage-driven inflammation. This approach allowed us not only to establish correlative patterns between microbial taxa and psoriasis but also to mechanistically define a microbiota–metabolite–immune signaling pathway that underpins skin barrier homeostasis.

Genistein, a predominant isoflavone derived from soy-based foods, acts as a phytoestrogen capable of binding to estrogen receptors.^34^ ^,^ ^35^ It possesses potent antioxidant and anti-inflammatory properties and exerts dual regulatory effects on cellular homeostasis by promoting apoptosis and suppressing excessive proliferation. Building on dietary metabolites, we sought to refine the understanding of microbe–host interactions. Rather than viewing microbial metabolites through a broad, descriptive lens, we reframed the model into a more precise microbe–enzyme–metabolite axis that directly shapes immune regulation. Our work addresses this critical knowledge gap by not only identifying a significant depletion of *P. distasonis* in both psoriatic patients and preclinical models but also by providing a direct causal pathway: *P. distasonis* metabolizes dietary isoflavones via its β-glucosidase activity to produce genistein, which subsequently suppresses skin inflammation. Importantly, this protective effect relies predominantly on robust, transient enzymatic biotransformation within the gut lumen rather than stable colonization. These insights suggest that targeted interventions for psoriasis could include engineered probiotic strains or dietary strategies designed to enhance the production of specific metabolites, such as genistein. Beyond psoriasis, this framework may also be relevant to other immune-mediated disorders where checkpoint signaling and barrier integrity lie at the heart of disease pathogenesis.

Macrophages play a crucial role in regulating systemic immune diseases, including psoriasis and inflammatory bowel disease (IBD), where modulation of macrophage polarization significantly impacts the inflammatory response.^36^ ^,^ ^37^ While psoriasis is traditionally viewed as a T-cell-driven disease characterized by dysregulated IL-23/IL-17 signaling, our findings complement this paradigm by identifying a gut microbiota-derived metabolic pathway that regulates innate immune activation upstream of the adaptive immune response. Specifically, we demonstrate that *P. distasonis*-derived genistein suppresses macrophage inflammatory activation through the CD200–CD200R axis, thereby limiting NF-κB signaling and promoting skin homeostasis. Notably, blockade of CD200 largely abolished the protective effects of genistein, supporting the functional importance of this pathway in mediating gut–skin communication. Nevertheless, the contribution of other immune populations, including T cells, dendritic cells, and neutrophils, remains to be determined. In addition, although our *in vitro* studies utilized LPS-stimulated macrophages and the in vivo psoriasis model was induced by the TLR7 agonist IMQ, CD200–CD200R signaling has been reported to broadly suppress myeloid cell activation through convergent NF-κB-dependent pathways. Future studies are warranted to define the cellular source of CD200 in psoriatic skin, clarify the applicability of this mechanism across distinct inflammatory contexts, and identify the upstream molecular target through which genistein activates the CD200–CD200R axis.

Despite the robustness of our findings, this study has several limitations that warrant consideration. First, although our cross-sectional analysis revealed a significant depletion of *Parabacteroides distasonis* in the psoriatic gut microbiome, the functional consequence of this dysbiosis—specifically, an alteration in genistein bioavailability—remains to be directly demonstrated in patients. Therefore, prospective clinical trials that co-assess microbial signatures, metabolomic shifts, and validated psoriatic endpoints are critically required. Such studies represent the definitive step needed to translate our proposed microbe-metabolite axis from a laboratory model to a clinically actionable therapeutic strategy. Second, while we identified genistein as a key active metabolite, *P. distasonis* likely produces a consortium of other bioactive molecules, such as short-chain fatty acids (SCFAs) and secondary bile acids, which could act synergistically to promote immune homeostasis. A multi-omics approach in future work could deconvolve these complex interactions.

In summary, this work identifies *P. distasonis*–derived genistein as a key mediator of gut microbiota–macrophage crosstalk in psoriasis, providing mechanistic support for dietary and probiotic strategies targeting the gut-skin axis. By linking microbial metabolism to macrophage polarization and cytokine signaling, our findings pave the way for novel combinatorial therapies that integrate isoflavone supplementation, microbiome modulation, and immunomodulation to ameliorate psoriatic inflammation. However, the clinical significance of targeting the gut-skin axis in psoriasis management warrants further investigation.

## Materials and methods

### Animal study

Male C57BL/6 mice (6–8 weeks old, weighing 19–23 g) were purchased from the Guangdong Animal Experimental Center. For the germ-free (GF) experiments, age- and sex-matched GF C57BL/6 mice were strictly housed and maintained in sterile flexible film isolators. All animal experiments were approved by the Animal Care Committee of the Southern Medical University of China. The mice were housed under the following controlled conditions: a steady temperature of 20 ± 2 °C, a 12 h light/12 h dark cycle, and ad libitum access to aseptic food and water. The mouse model of psoriasis was established as described in a previous publication. The experimental design is illustrated in the figure. Specifically, the mice were randomly divided into different groups. The back of C57BL/6 mice was shaved one day before induction. Then, 62.5 mg of cream containing 5% Imiquimod (IMQ) (Sichuan MingXin Pharmaceutical, China) was evenly applied on the back daily for seven days. Genistein (MedChemExpress, USA) was administered intraperitoneally once daily during the first three days of IMQ induction. The mice were euthanized at the end of the experiment, and skin, feces, and blood samples were collected for further analysis.

### Fecal microbiota depletion and transplantation experiment (FMT)

Fecal samples were collected and resuspended in 0.125 g/mL PBS. Mice were pretreated with antibiotics (200 mg/kg neomycin sulfate, 200 mg/kg metronidazole, 200 mg/kg ampicillin, and 100 mg/kg vancomycin) via oral gavage for 5 days, followed by the administration of 0.2 mL fecal suspension from either healthy or IMQ-group mice for 7 days. Post-transplant recipient mice were then subjected to topical treatment with imiquimod.

## 16S rRNA gene sequencing analysis

Before euthanizing mice after 7 days of intervention, fecal samples were collected from each mouse and placed in individual Eppendorf tube using sterile forceps (Greiner, China) and stored in saline at −80 °C to collect fecal microbial samples. Fecal microbial DNA was isolated using the DNeasy PowerSoil Kit (QIAGEN, Germany) following the manufacturer’s instructions. The V-region of the 16S rRNA gene was amplified via PCR using Pyrobest DNA Polymerase (TaKaRa, Japan). Target amplicons were purified from agarose gels with the AxyPrep DNA Gel Extraction Kit (Axygen, USA). Following quantification using the Quant-iT PicoGreen dsDNA Assay (Invitrogen, USA), sequencing libraries were constructed in accordance with the Illumina TruSeq protocol. Bioinformatic processing was executed via the QIIME2 (version 2019.4) pipeline, incorporating the DEMUX and Cutadapt plugins for demultiplexing and primer removal.^38^ ^,^ ^39^ Sequence denoising and chimera elimination were performed using DADA2. The sequencing procedures and analysis parameters were adapted from established protocols. α-diversity metrics, including Chao1, observed species, Shannon, Simpson, Faith pd and Goods converage, as well as beta-diversity metrics, were estimated using the diversity plugin. The raw 16SrRNA sequencing data had been uploaded to the NCBI public database under the accession number PRJNA1201600.

### Metabolomics analysis

Fecal contents were collected from mice. Fecal samples were homogenized in 90% ultrapure water via sonication, followed by metabolic extraction using cold methanol. Post-centrifugation (13,000 rpm, 10 min, 4 °C), the supernatants were analyzed using LC-ESI-MS. For positive mode, the mobile phase comprised 0.1% formic acid in acetonitrile (B2) and water (A2). The gradient started at 8% B2 (0–1 min), increased to 98% B2 (1–8 min), held for 2 min, and returned to 8% for equilibration. Negative mode utilized acetonitrile (B3) and 5 mM ammonium formate (A3) with a comparable elution profile. Data processing was facilitated by Compound Discoverer 3.1 software (Thermo Scientific).

### Dermatoscope, skin ultrasound and transepidermal water loss rates (TEWL)

Cutaneous inflammation and physiological parameters were evaluated using the SkinLab Z5010115 system (Cortex Technology, UK). Transepidermal water loss (TEWL) and macroscopic skin changes were recorded to assess barrier dysfunction. Furthermore, high-resolution ultrasonography was employed to visualize tissue architecture; the reflected signals indicated the density of different skin layers, with the epidermis and dermis showing distinct reflectivity patterns. The epidermis exhibited high reflectivity, appearing white or pale yellow, while the dermis displayed a mixture of various colors. Subcutaneous fat and muscle fibers showed low-intensity signals (depicted as deep green and black).

### Measurement of isoflavones levels

Mouse fecal specimens were homogenized with steel beads in 100% (w/v) methanol (2 min, 60 Hz) followed by centrifugation (15 min, 14,000 rpm, 4 °C). For bacterial supernatants, *P. distasonis* (1 × 10^8^ CFU/mL) was cultured with genistin (50μm) at 37 °C anaerobically for 24 h, 100 μL aliquots were vortex-mixed with 900 μL methanol and centrifuged under identical conditions. Supernatants were subjected to flavonoid aglycone extraction using 1 mL ethyl acetate (Macklin, Shanghai, China). After phase separation, 800 μL organic layers were evaporated to dryness under nitrogen and reconstituted in 100 μL methanol (Macklin) prior to analysis. Chromatographic separation was achieved on a Shim-pack GIST C18-AQ column (Shimadzu) using Nexera LC-30A UHPLC system with mobile phase A (0.1% aqueous formic acid) and B (methanol). The gradient program was: 10–100% B (0–5 min), 100% B (5–8 min), 10% B (8–11 min) at 0.4 mL/min. MS detection employed Shimadzu LCMS-8050 triple quadrupole mass spectrometer with dual ESI polarity switching. Isoflavones were quantified via external calibration curves generated from authentic standards, with concentrations determined by peak area correlation.

### Bacterial culture

*P. merdae* and *P. distasonis* which purchased from *American Type Culture Collection* (*ATCC, Manassas, VA*) were both cultured anaerobically in brain heart infusion (BHI) (Hopebio, Qingdao, China) medium at 37 °C. *P. distasonis* was incubated with 100 μM conduritol B epoxide (CBE) under anaerobic conditions at 37 °C for 24 h, hereafter designated as *P. distasonis*-CBE. *Escherichia coli Nissle 1917* was acquired from MiaoLingBio (Wuhan, China) and maintained under anaerobic conditions in Luria-Bertani (LB) medium at 37 °C. Microbial precipitates were acquired via centrifugation at 5000 × g for 5 min and then diluted to 1 × 10^9^ CFU in phosphate buffer saline for gavage administration to mice.

To evaluate the specific functional roles of distinct *Parabacteroides* species, mice were subjected to oral gavage with *P. distasonis*, *P. merdae*, or *P. goldsteinii*. Notably, no antibiotic pretreatment was administered prior to the bacterial intervention. This design was deliberately chosen to mimic a real-world, prophylactic probiotic supplementation scenario, allowing us to assess the barrier-protective effects of these exogenous strains within an intact, conventional gut microbiota. The bacterial pellets were harvested by centrifugation at 5000 × g for 5 min and resuspended in phosphate-buffered saline (PBS) to a final dose of 1 × 10^9^ CFU per mouse for daily oral administration.

### The *E. coli*-bglX construction

The construction of the *E. coli*-bglX engineered strain was conducted following the experimental methodology described by *Hong et al*. Briefly, a dual-plasmid CRISPR-Cas9 system was employed to engineer *E. coli* strains expressing β-glucosidase (β-GC). The β-glucosidase-encoding gene was PCR-amplified from *P. distasonis* genomic DNA, while the oxygen-responsive promoter P_*nirB*_ was isolated from *E. coli*. CRISPR-mediated homologous recombination facilitated precise integration of the β-GC expression cassette into the chromosomal attλ site of E. coli. Transformants were selected on LB agar plates supplemented with 50 μg/mL kanamycin and 25 μg/mL chloramphenicol. Gene-specific amplification was achieved using primer pair F (5′-ATGAACAAACTATTAATGGGCGC-3′) and R (5′-CTATCTTTTCAGAAGATCGATCCTCTC-3′), designed with 30-bp homology arms for seamless recombination.

### qRT-PCR analysis

Total RNA was extracted from mouse tissues using TRIzol agent (Vazyme, Nanjing, China) according to the manufacturer's protocol. Reverse transcription was performed to obtain cDNA (20 ng), which served as a template for real-time PCR using TB Green™ Premix Ex Taq™ II (Takara Bio, Japan) on a Bio-Rad 7500 system. Gene expression levels were calculated using the 2^−ΔΔCt^ method, with 18S rRNA serving as the internal normalization control.Specific primer sequences are listed in Table 1.

**Table 1.** The sequences of real-time PCR primers.

Gene name	Forward 5'- 3'	Reverse 5'- 3'
*Il-17*	TCAGCGTGTCCAAACACTGAG	CGCCAAGGGAGTTAAAGACTT
*Il-23*	CAGCAGCTCTCTCGGAATCTC	TGGATACGGGGCACATTATTTT
*Tnfa*	CACCACGCTCTTCTGTCTACTG	GGGCTACGGGCTTGTCACTC
*Il-6*	CTGTTGTGGGTGGTATCCTCTGT	TTGCCTTCTTGGGACTGATGTT
*Il-1b*	GCCCGTCCTCTGTGACTCGT	TGTCGTTGCTTGTCTCTCCTTGTA
*Nos2*	GCAAACATCACATTCAGATCCC	TCAGCCTCATGGTAAACACG
*MCP-1*	CCACTACCTTTTCCACAACC	GTCCGAGTCACACTAGTTCA
*Cd200*	GCCAGAGTTTCCACACCTTC	TCCATCTCCACGATGTTTGC
*Gapdh*	AGTGCCAGCCTCGTCTCATAGA	GCCTTGACTGTGCCGTTGAACT

### Cell culturing

Primary mouse bone marrow-derived macrophages (BMDMs) were harvested from the femurs and tibias of C57BL/6 mice. Briefly, the bone marrow cavity was flushed with ice-cold DMEM (Gibco, USA), and the resulting cell suspension was passed through a 70-μm cell strainer to remove tissue aggregates. After centrifugation and resuspension, the nucleated cells were adjusted to a concentration of 1 × 10^6^/mL. For macrophage differentiation and polarization, cells were cultured in DMEM supplemented with 5% fetal bovine serum (FBS; Gibco), 1% penicillin/streptomycin, 20 ng/mL recombinant mouse GM-CSF (Sino Biological, China), and 20 ng/mL recombinant IL-4 (Miltenyi Biotec, Germany). The cultures were maintained at 37 °C in a humidified atmosphere containing 5% CO_2_ for a duration of 7 days to ensure full maturation.

### Histology and immunohistochemistry assays

For histological evaluation, dorsal skin tissues were immersion-fixed in 4% paraformaldehyde and subsequently embedded in paraffin wax. Sections (5-μm thick) were prepared using a microtome and stained with hematoxylin and eosin (H&E; Servicebio, China) to assess pathological changes. For immunohistochemical (IHC) analysis, tissue sections underwent a systematic process involving deparaffinization, heat-induced antigen retrieval, and permeabilization with 0.25% Triton X-100. To minimize non-specific binding, sections were incubated with 5% bovine serum albumin (BSA) for blocking. Primary antibodies against loricrin (1:400; Proteintech, China) and filaggrin (1:400; Abcam, UK) were applied to evaluate skin barrier markers, followed by incubation with a goat anti-rabbit IgG secondary antibody (Proteintech, China) to visualize immunoreactivity.

### Flow cytometry

Skin cell suspensions were prepared from IMQ-treated mice. First, dorsal skin harvested from IMQ-induced mice was subjected to enzymatic digestion using collagenase IV for 45 min at 37 °C. The resulting cell homogenate was resuspended in ice-cold PBS supplemented with 2% fetal bovine serum (FBS) and passed through a 100-μm sterile nylon mesh to remove debris. Lymphoid cell populations were further enriched via density gradient centrifugation using a 40%/70% Percoll interface. For immunophenotyping, the isolated cells were incubated with APC-conjugated anti-F4/80 and FITC-conjugated anti-CD11b antibodies (Biolegend, USA) for 30 min in the dark. Flow cytometric acquisition was performed on a BD LSRFortessa system, and the captured data were processed using FlowJo software (version 10; BD Biosciences, USA).

### Western blot analysis

The experiment was carried out in accordance with a previously described protocol(references). Briefly, skin tissues were lysed in RIPA Lysis Buffer (Thermo Scientific, MA, USA). The protein concentration was quantified using a BCA kit (Thermo Scientific, MA, USA). Next, proteins were separated by SDS-PAGE and transferred into nitrocellulose membranes (Merck, MA, USA), followed by blocking with 5% bovine serum albumin (BSA) (Thermo Scientific, MA, USA). They were then incubated with the primary antibodies, anti-β-actin (1:10000, 4967S, Cell Signaling Technology, USA), anti-CD200 (1:1000, DF10155, Affinity), anti-*p*-65 (1:1000, 8242 T, Cell Signaling Technology, USA), anti-*p*-p65 (1:1000, 3033 T, Cell Signaling Technology, USA), anti-IKBα (1:1000, 61294S, Cell Signaling Technology, USA), and anti-*p*-IKKα/β (1:1000, 2859 T, Cell Signaling Technology, USA), anti-filaggrin (1:1000, DF13653, Affinity, USA), anti-k10 (1:2000, YB33422, Signalway Antibody, USA) overnight at 4 °C and then incubated with the corresponding secondary antibody (1:10000, Cell Signaling Technology, USA) for 1 hour at room temperature. Enhanced chemiluminescence (ECL, Biosharp, Beijing, China) was then used to visualize protein bands.

### Statistical analysis

Statistical analyses were conducted using GraphPad 9.0 and SPSS 22.0. Data were presented as the mean ± standard deviation. Normal distribution and similar variance between groups were assessed. Group differences were evaluated using unpaired Student's t-test or Holm-Sidak One way ANOVA for post hoc tests (unpaired, two-tailed) (*P < 0.05, **P < 0.01, ***P < 0.001, ****P < 0.0001). Graphs were generated using BioRender.

## Supplementary Material

Supplementary Material Supplement_figures.docx

## Data Availability

The raw 16SrRNA sequencing data had been uploaded to the NCBI public database under the accession number PRJNA1201600. Other derived data supporting the findings of this study are available from the corresponding author [Xuan Wang] on request.
