# Supplementary material for: Gut-derived genistein from Parabacteroides distasonis alleviates psoriatic inflammation via CD200-mediated NF-κB inhibition in mice
Source: Gut Microbes. 2026 Jul 16;18(1):2701386. doi: 10.1080/19490976.2026.2701386 (PMC13378712; doi:10.1080/19490976.2026.2701386)
Supplement: Supplementary Material — Supplement_figures.docx [file KGMI_A_2701386_SM3755.docx]

**Supplement figures and table**


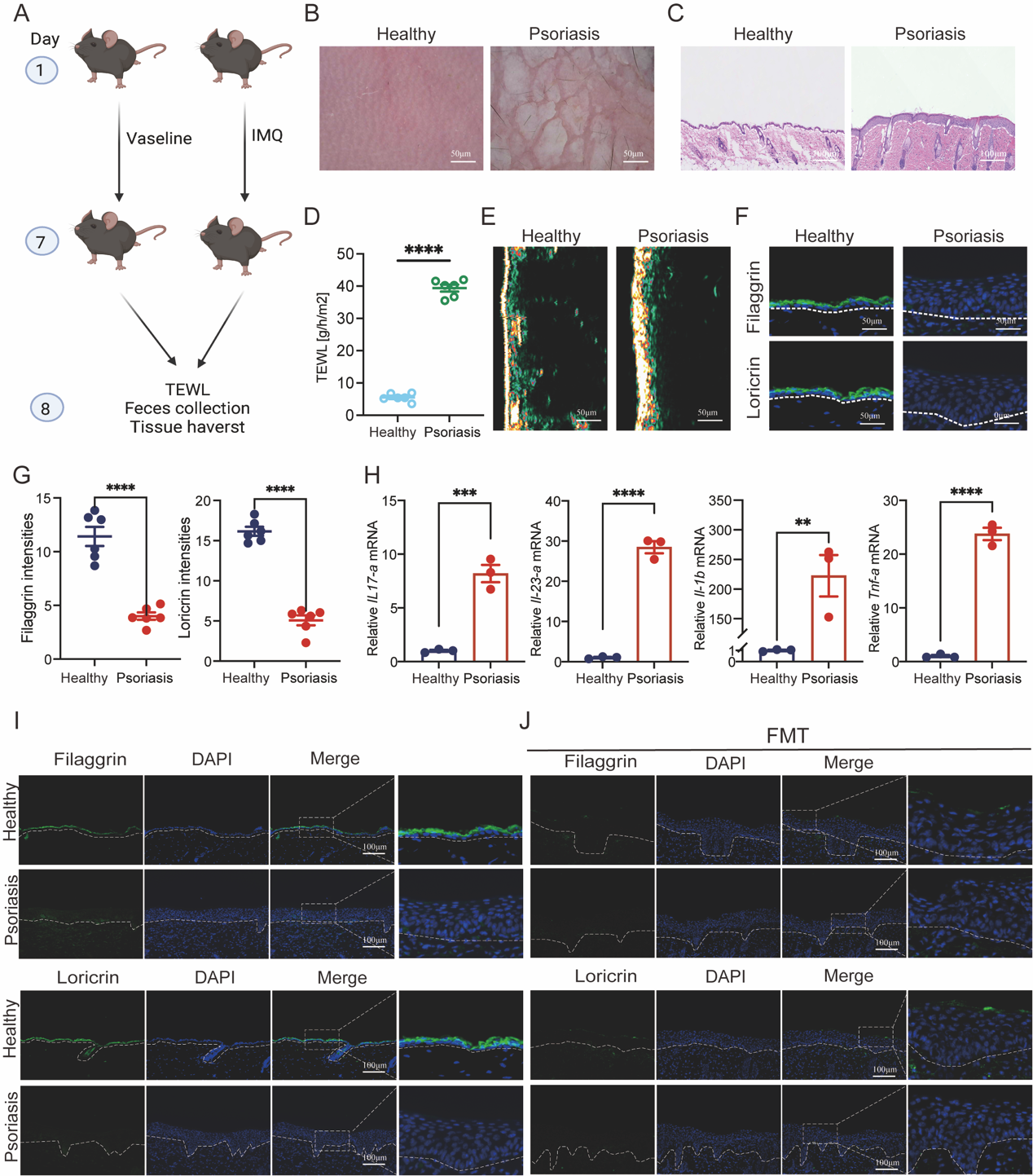


**Figure S1: Assessment of skin barrier function in IMQ-induced psoriatic mice, related to Figure 1.** (A) Experimental design of IMQ-induced psoriatic mouse model (n=6 per group). (B) Dermatoscopic evaluation of skin lesions post-IMQ induction. (C) H&E staining following IMQ treatment. (D) TEWL measurements after IMQ treatment. (E) Ultrasonographic examination of skin thickness in IMQ-treated mice. (F, G, I) Immunofluorescence staining of skin tissues and quantitative analysis following IMQ treatment (n=6 per group). (H) qRT-PCR analysis of inflammatory cytokine mRNA levels (*Il-17, Il-23, Il-1β, Tnf-α*) in IMQ-treated skin (n=3 per group). (J) Ultrasonographic evaluation of epidermal structure in psoriatic mice following FMT intervention. **P<0.01, ****P<0.0001.


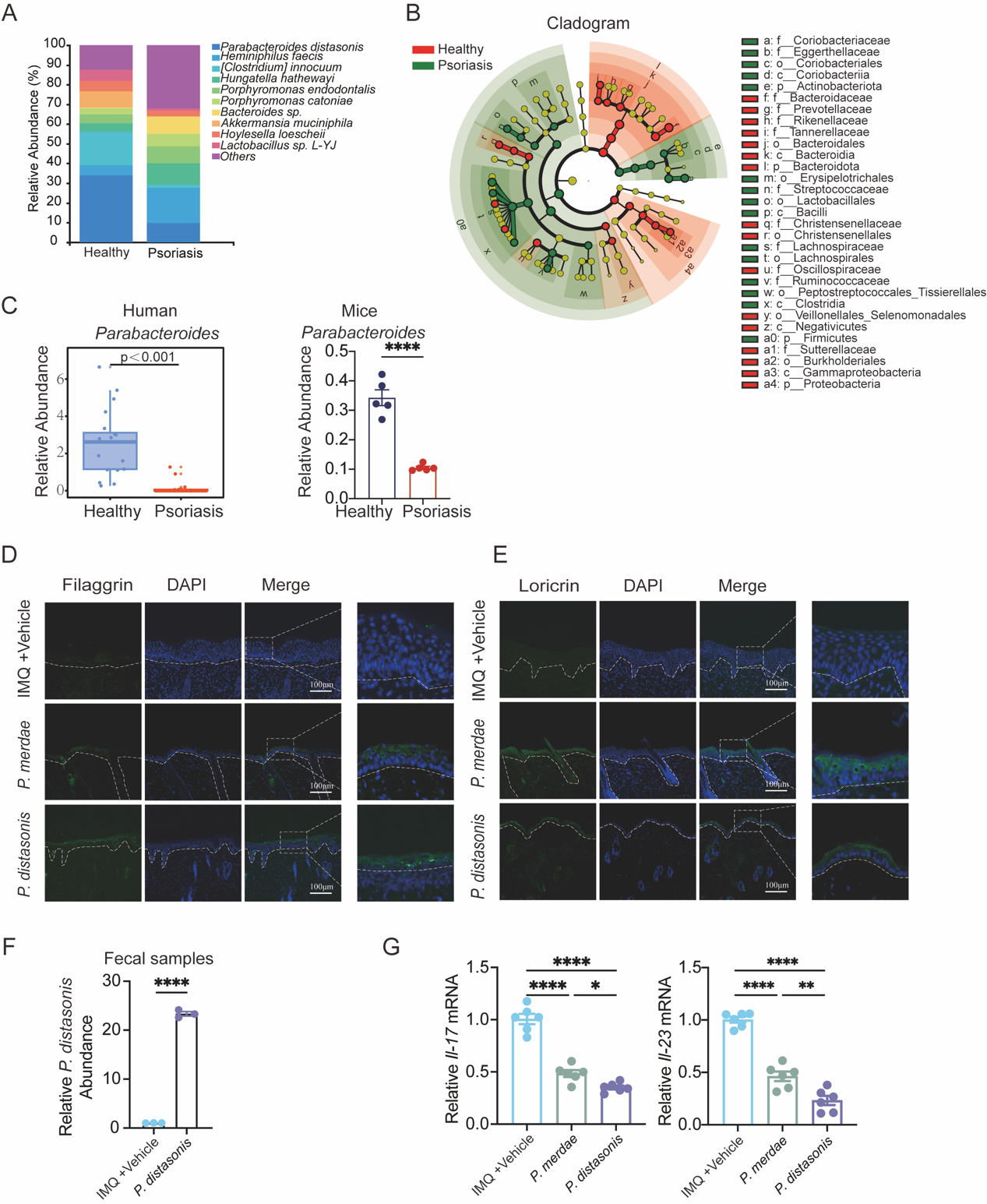


**Figure S2: Exogenous administration of *P. distasonis* improves epidermal permeability barrier function,** **related to Figure 2.** (A) Species-level taxonomic profiling of gut microbial communities in psoriatic mice (n=5 per group). (B) Phylogenetic cladogram illustrating gut microbes’ alterations in human psoriatic cohorts. (C) Differential abundance of *Parabacteroides* genus in fecal samples from psoriasis patients and mice. (D, E) Immunofluorescence analysis of skin sections following therapeutic intervention with *P. distasonis* and *P. merdae* strains. (F) Detection of fecal *P. distasonis* abundance following colonization by *P. distasonis.* (G) qRT-PCR analysis of inflammatory cytokine mRNA levels (*Il-17, Il-23*) following treatment (n=6 per group). ns, P>0.05; *P<0.05; **P<0.01; ****P<0.0001.


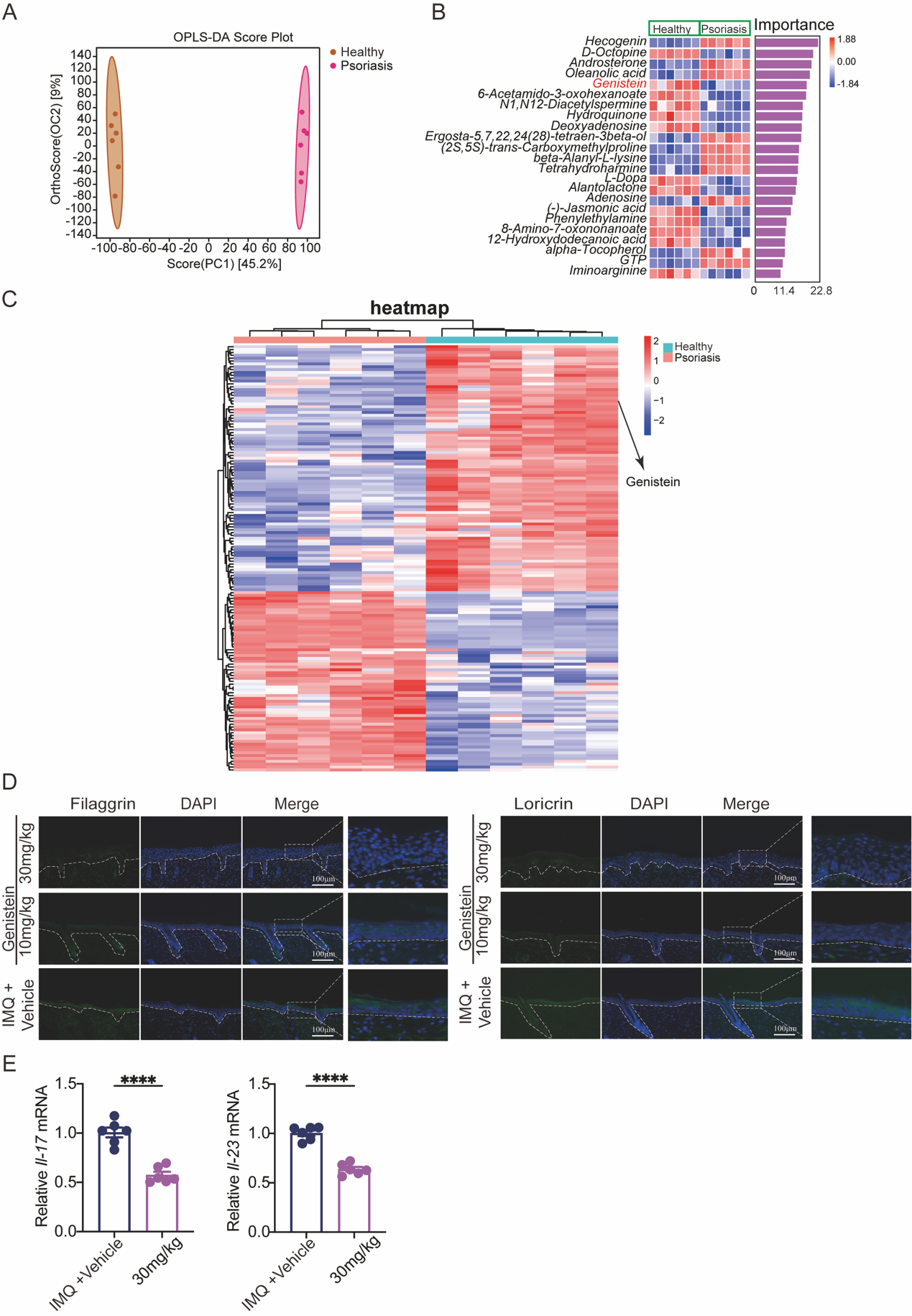


**Figure S3:** **Genistein improves epidermal permeability barrier function in IMQ-treated mice, related to Figure 3.** (A) OPLS-DA score plot of fecal metabolomics (n=6 per group). (B) Machine learning-driven fecal metabolomic profiling (n=6 per group). (C) Heatmap visualization of genistein abundance in fecal metabolites (n=6 per group). (D) Immunofluorescence images of skin sections following genistein intervention. (E) qRT-PCR analysis of inflammatory cytokine mRNA levels (*Il-17, Il-23*) following treatment (n=6 per group). ****P<0.0001.


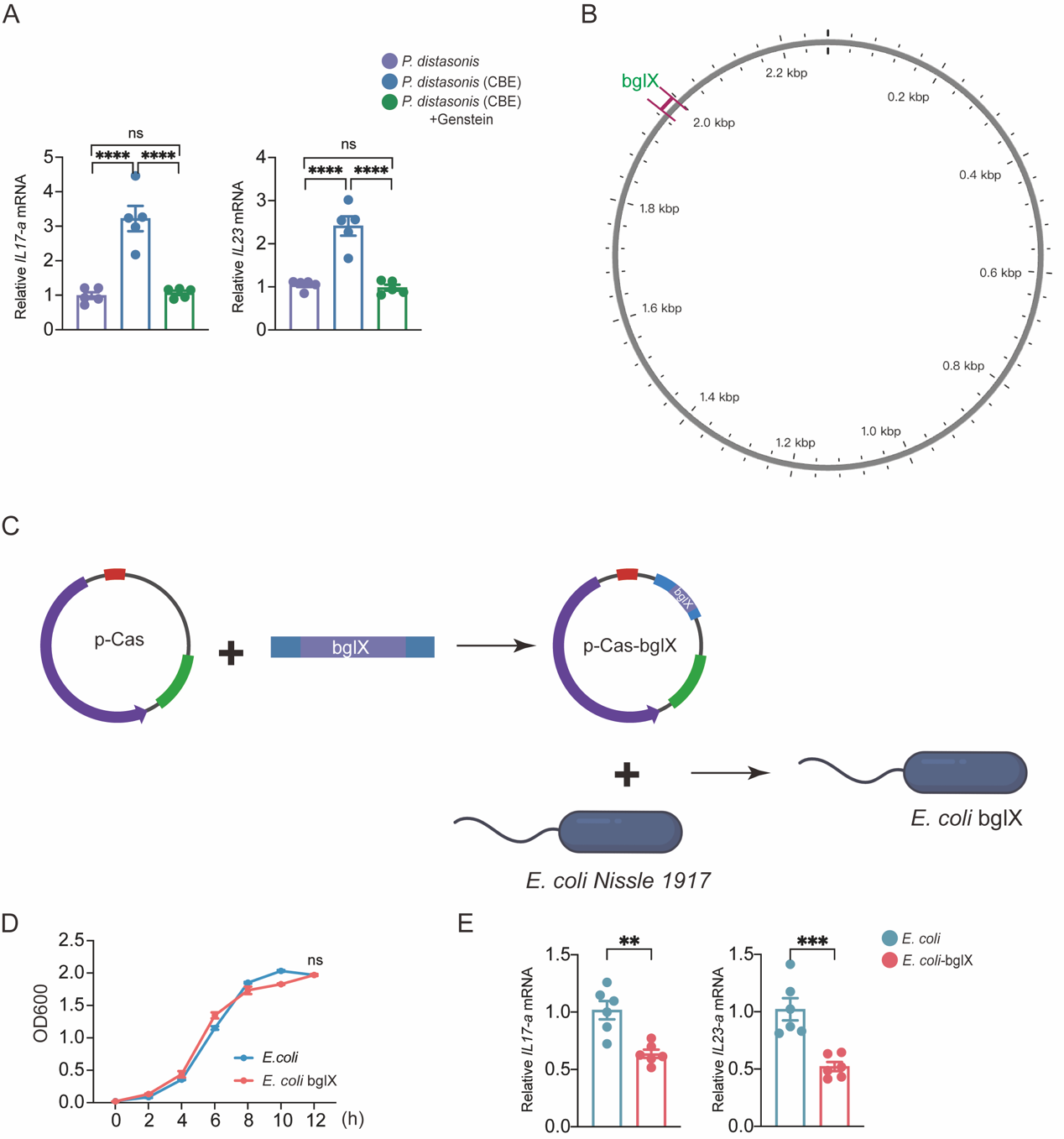


**Figure S4: β-glucosidase mediates the genistein-dependent protective effects of *P. distasonis*, related to Figure 5.** (A) Genomic locus of bgl-X gene in the complete genome sequence of *P. diatasonis* (n=5 per group). (B) Quantitative analysis of *Il-17* and *Il-23* mRNA expression levels in dorsal skin of *P. distasonis* with or without CBE pretreatment (n=5 per group). (C) Construction of β-glucosidase-overexpressing recombinant *E. coli Nissle 1917* strain (*E. coli*-bglX). (D) Growth curves of *E. coli*-bglX and wild-type *E. coli* after 12-hour cultivation. (E) Comparative mRNA expression profiles of *Il-17* and *Il-23* in dorsal skin following treatment with wild-type *E. coli* or *E. coli-*bglX strains (n=6 per group). ns, P>0.05; **P<0.01; ***P<0.001; ****P<0.0001.


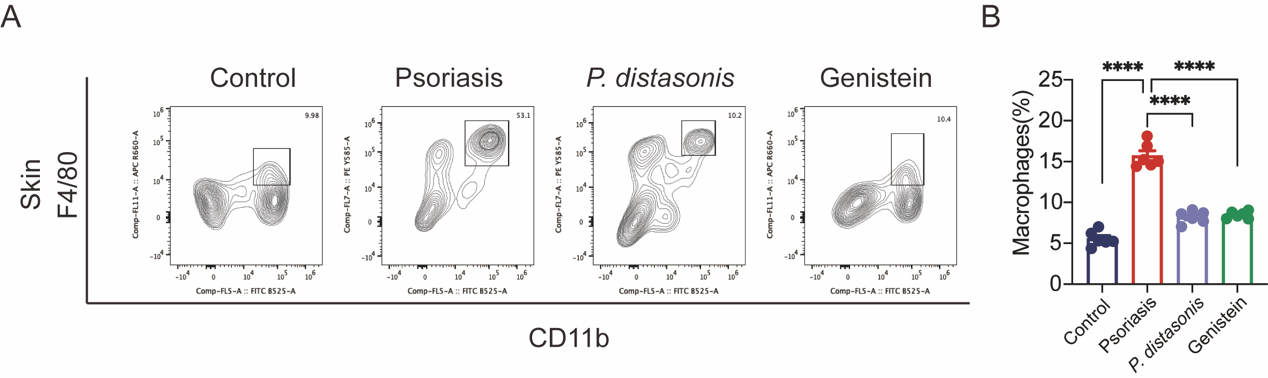


**Figure S5: Genistein attenuates macrophage-mediated inflammation by upregulating CD200, related to Figure 6.** (A, B) Flow cytometric quantification of skin macrophages in psoriatic mice (n=6 mice per group). ****P<0.0001.
